# Trends in AIDS-Defining Opportunistic Illnesses Incidence over 25 Years in Rio de Janeiro, Brazil

**DOI:** 10.1371/journal.pone.0098666

**Published:** 2014-06-05

**Authors:** Lara Coelho, Sandra Wagner Cardoso, Rodrigo Teixeira Amancio, Ronaldo Ismério Moreira, Dayse Pereira Campos, Valdiléa Gonçalves Veloso, Beatriz Grinsztejn, Paula Mendes Luz

**Affiliations:** Instituto de Pesquisa Clínica Evandro Chagas, Fundação Oswaldo Cruz, Rio de Janeiro, Brasil; McGill University AIDS Centre, Canada

## Abstract

**Objectives:**

To assess the temporal trends in incidence of AIDS-defining opportunistic illnesses in an urban cohort of a middle-income country.

**Methods:**

HIV infected patients aged ≥18 years at cohort entry were included in this analysis. We calculated incidence rates per 1000 persons-years of observation for the first opportunistic illness presented after cohort enrollment, from 1987 to 2012. Trends for overall and specific opportunistic illnesses were tested and incidence rate ratios for the most recent calendar period were calculated as the ratio between the incidence rate observed in the most recent period of the study (2009–2012) and the incidence rate observed in first period of the study (1987–1990).

**Results:**

Overall, 3378 patients were included in this analysis; of which 1119 (33%) patients presented an opportunistic illness during follow up. Incidence rates of all opportunistic illnesses decreased over time, and the overall opportunistic illness incidence rates fell from 295.4/1000 persons-years in 1987–1990 to 34.6/1000 persons-years in 2009–2012. Tuberculosis, esophageal candidiasis, cerebral toxoplasmosis and *Pneumocystis jirovecii* pneumonia were the most incident opportunistic illnesses in the cohort. Tuberculosis had the highest incidence rate in the study period. The peak in tuberculosis incidence occurred in 1991–1993 (80.8/1000 persons-years). Cerebral toxoplasmosis was the third most incident opportunistic illness in the study, with a peak of incidence of 43.6/1000 persons-year in 1987–1990.

**Conclusions:**

All opportunistic illnesses incidence rates decreased over the years but they still occur in an unacceptable frequency. Tuberculosis co-infection among HIV-infected persists as an important challenge for health care professionals and policy makers in our setting. Impressively high rates of cerebral toxoplasmosis were found suggesting that its incidence among HIV-infected is linked to the high prevalence of *Toxoplasma gondii* infection in the general population.

## Introduction

Major advances have been achieved in the management and treatment of HIV infected patients. As result the incidence and morbimortality of opportunistic illnesses have dramatically declined in the years that followed combination antiretroviral therapy (cART) introduction [9], [17], [31], [35]. Nonetheless, opportunistic illnesses (OI) still represent a major cause of death and hospitalization in HIV infected patients, in these settings [5], [30], [34], [36].

The incidence of specific OI in HIV infected patients can vary geographically mainly as a result of the differential burden of tropical infectious diseases observed in middle-low income settings [11], [47]. One prominent example is tuberculosis with only 22 countries located in Africa, Asia and Latin America (including Brazil) being responsible for more than 80% of all cases worldwide [45].

Brazil is a middle-income country where publically funded universal access to cART, laboratory monitoring with CD4 cell counts and viral load quantification and OI prophylaxis and treatment have been provided since 1996 [29]. Yet, few studies have evaluated trends in the incidence of OI in our setting [11].

In this study, we examine rates and patterns of OIs incidence in an urban clinical cohort in Rio de Janeiro, Brazil, from 1987 to 2012.

## Patients and Methods

### Ethics Statement

This study was approved by the ethics committee of the Evandro Chagas Clinical Research Institute of the Oswaldo Cruz Foundation (CAAE 0032.0.009.000-10) and was conducted according to the principles expressed in the Declaration of Helsinki. All patient records/information was de-identified prior to analysis.

### Study Population

Evandro Chagas Clinical Research Institute (IPEC/Fiocruz) is a reference center for treatment of patients with HIV/AIDS, in Rio de Janeiro, Brazil. As of June 2013, over 5,000 patients have been cared for at IPEC. A longitudinal, observational, clinical database has been maintained on patients receiving care at IPEC since 1986. Cohort procedures have been described and results published [18], [19], [27]. The database includes socio-demographic, laboratory, clinical, and therapeutic information abstracted from the medical records and is updated periodically by trained staff.

For this study we included all patients 18 years of age or older at cohort entry, who were followed for a period of at least 60 days from 1 January 1987 to 31 October 2012. The start of the observation period was defined based on the first medical appointment at IPEC. Follow-up ended at the date of the first OI for those who presented with an OI during follow-up. For those patients who never experienced an OI during follow-up, the end of follow-up was defined as the date of the last clinic visit, the date of death or study closure, whichever occurred first.

**Table 1:** Characteristics of patients included in the opportunistic illnesses incidence analyses by period of cohort enrollment, IPEC cohort, period 1987–2012.

	1987–1990	1991–1993	1994–1996	1997–1999	2000–2002	2003–2005	2006–2008	2009–2012	p-value
	(n = 186)	(n = 291)	(n = 205)	(n = 243)	(n = 369)	(n = 427)	(n = 933)	(n = 1124)	
**Age at enrollment years** (median, IQR)	31.9 (28.5,39)	34.7 (28.6,40.6)	33 (28.4,38.8)	33.5 (28.4,38.9)	36.6 (29.6,42.5)	37.1 (30.3,43.2)	35.4 (28.7,43.1)	34.5 (27.9,42.3)	<0.001
**Male sex**	161 (86.6)	210 (72.2)	113 (55.1)	114 (46.9)	225 (61)	306 (71.7)	615 (65.9)	770 (68.5)	<0.001
**Non white race/ethnicity**	63 (33.9)	128 (44)	72 (35.1)	106 (43.6)	143 (38.8)	155 (36.4)	467 (50.2)	653 (58.8)	<0.001
**Educational level**									0.198
0–8 years	78 (47.6)	152 (52.2)	119 (58)	136 (56)	187 (50.8)	208 (48.7)	469 (50.4)	546 (49.1)	
9+ years	86 (52.4)	139 (47.8)	86 (42)	107 (44)	181 (49.2)	219 (51.3)	462 (49.6)	565 (50.9)	
**HIV exposure category**									<0.001
Heterossexual	31 (16.7)	126 (43.3)	118 (57.6)	140 (57.6)	209 (56.6)	208 (48.7)	525 (56.3)	502 (44.7)	
MSM	126 (67.7)	127 (43.6)	61 (29.8)	66 (27.2)	118 (32)	168 (39.3)	324 (34.7)	452 (40.2)	
IDU	15 (8.1)	20 (6.9)	10 (4.9)	7 (2.9)	6 (1.6)	3 (0.7)	13 (1.4)	9 (0.8)	
Other/Unknown	14 (7.5)	18 (6.2)	16 (7.8)	30 (12.3)	36 (9.8)	48 (11.2)	71 (7.6)	161 (14.3)	
**Median baseline CD4** (IQR)	172 (96,225)	251.5(110.5,674.8)	335.5(149.2,499.8)	323.5(148.2,500.5)	220(96.2,390)	268(103.2,464.8)	311.5(112.8,524.5)	310(107.5,551)	0.009
0–49 cells/mm^3^	1 (0.5)	3 (1)	3 (1.5)	14 (5.8)	25 (6.8)	49 (11.5)	94 (10.1)	126 (11.2)	<0.001
50–199 cells/mm^3^	6 (3.2)	9 (3.1)	9 (4.4)	34 (14)	69 (18.7)	67 (15.7)	150 (16.1)	191 (17)	
200–349 cells/mm^3^	2 (1.1)	8 (2.7)	5 (2.4)	23 (9.5)	45 (12.2)	63 (14.8)	137 (14.7)	155 (13.8)	
350+ cells/mm^3^	2 (1.1)	14 (4.8)	17 (8.3)	63 (25.9)	61 (16.5)	115 (26.9)	331 (35.5)	391 (34.8)	
Missing	175 (94.1)	257 (88.3)	171 (83.4)	109 (44.9)	169 (45.8)	133 (31.1)	221 (23.7)	261 (23.2)	
**Baseline VL**									<0.001
0–399 copies/mm^3^	0 (0)	0 (0)	0 (0)	10 (4.1)	11 (3)	33 (7.7)	66 (7.1)	125 (11.1)	
400–4,999 copies/mm^3^	0 (0)	1 (0.3)	0 (0)	20 (8.2)	20 (5.4)	27 (6.3)	45 (4.8)	81 (7.2)	
5,000–99,999 copies/mm^3^	0 (0)	0 (0)	1 (0.5)	51 (21)	77 (20.9)	101 (23.7)	177 (19)	296 (26.3)	
100,000+ copies/mm^3^	0 (0)	0 (0)	0 (0)	39 (16)	86 (23.3)	85 (19.9)	146 (15.6)	196 (17.4)	
Missing	186 (100)	290 (99.7)	204 (99.5)	123 (50.6)	175 (47.4)	181 (42.4)	499 (53.5)	426 (37.9)	
**OI at enrollment**	41 (22)	47 (16.2)	35 (17.1)	47 (19.3)	100 (27.1)	100 (23.4)	189 (20.3)	325 (28.9)	<0.001
**Median time from HIV diagnosis and cohort enrollment, days** (IQR)	0 (0,62.5)	33 (0,111)	58 (0,226)	104 (29.5,481.5)	123 (30,848)	260 (45,2370)	110 (24,742)	53 (8.8,574.5)	<0.001

OI: opportunistic illness; MSM: men who had sex with men; IDU: injection drug users; VL: viral load.

### Database Validation

To assess the quality of the OI diagnoses present in the IPEC longitudinal database, we performed a clinical validation of diagnoses by direct comparison of database with chart review. Ten percent of the patients who presented an OI during follow-up (n = 118 patients) were randomly selected for chart review and validation of the first and concomitant OI, that is, those occurring within 30 days of the date of the first OI.

A trained HIV medical specialist used two criteria to validate the diagnoses. The first, which classified diagnoses into certain, probable and possible, took into consideration all forms of complementary exams, including radiological, microbiological and hystopatological, in addition to medical prescriptions. This criterion had already been used by our team in other study [34]. The second criterion was built based on the case definition for HIV-related diseases established by the WHO [43]. According to this criterion, the diagnoses were classified either as definitive or clinical.

The validation showed that 95% of the OI diagnosis were confirmed using either criteria. Out of the confirmed diagnosis, 49% were classified as certain using the first criterion, and 56% were classified as definitive according to second (WHO) criterion.

Although WHO criteria for case definition of HIV-related diseases was applied for the database validation process described above, the selection of the opportunistic illnesses included in the incidence analysis were based on those present in the Center for Disease Control “1993 Revised Classification System for HIV Infection and Expanded Surveillance Case Definition for AIDS Among Adolescents and Adults” (CDC, 1993) [8].

### Outcome Measures

The outcome of interest was the occurrence of the first OI after cohort enrollment. Prevalent diagnosis at or up to 30 days of the date of cohort enrollment were excluded from the incidence rate calculation.

The opportunistic illnesses included in the analysis were those in the CDC 1993 definition [8], namely tuberculosis, esophageal candidiasis, toxoplasmosis cerebral, *Pneumocystis jirovecii* pneumonia (PCP), herpes simplex virus, cytomegalovirus disease, extrapulmonar cryptococcosis, Kaposi sarcoma, crypstosporidiosis, isosporosis, non tuberculosis mycobacterium disease, disseminated histoplasmosis, non Hodgkin lymphoma. We excluded from the analysis the following opportunistic illnesses: HIV associated encephalopathy, recurrent bacterial pneumonia, salmonella septicemia and wasting syndrome. The reasons for excluding these diseases were disease-specific, namely the low number of cases in the case of salmonella septicemia, the difficultly in establishing a diagnosis of recurrence in the case of bacterial pneumonia, and the subjective nature of the diagnosis criteria in the case of HIV associated encephalopathy and wasting syndrome.

### Statistical Methods

Eight calendar periods were defined a priori as follows: 1987–1990, 1991–1993, 1994–1996, 1997–1999, 2000–2002, 2003–2005, 2006–2008 and 2009–2012. Socio-demographic, behavioral, laboratory, clinical features were compared among all included patients according to date of cohort entry using Kruskal-Wallis test for continuous variables and Chi-square for categorical variables.

We estimated the incidence rates of the first OI in each calendar period. The incidence rate of an OI was defined as the ratio between the number of cases of a specific illness and the number of person-years at risk during that period. Person-years at risk were calculated for each patient as the sum of days at risk by period. Rates were calculated by 1000 person-years with respective 95% confidence intervals (CI) using a Poisson regression model. Trends for overall and specific OI were tested for all OI with frequency above 40 cases during the study period. Incidence rate ratios as a function of calendar period were calculated as the ratio between the incidence rate observed in the most recent period of the study (2009–2012) and the incidence rate observed in first period of the study (1987–1990).

## Results

**Figure 1:**
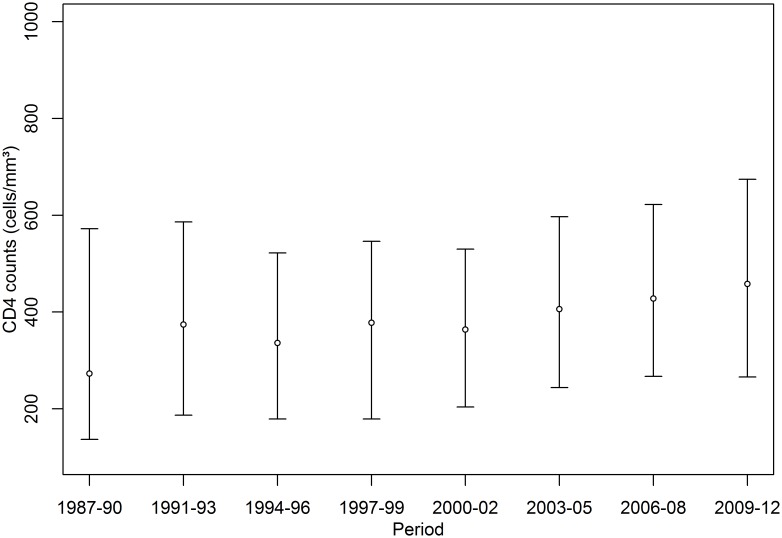
CD4 cell counts of patients under follow-up by calendar period: for each period medians and interquartile ranges are displayed.

**Figure 2:**
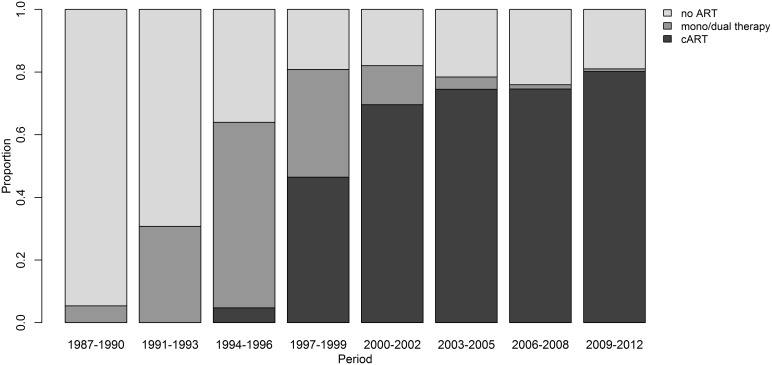
Use of antiretroviral therapy by calendar period.

From January 1^st^ 1987 to December 31^st^ 2012 a total of 3378 patients met the study criteria and were included in this analysis, 1119 (33%) presented an OI during follow-up. Age at enrollment increased over the years and male sex predominated in all of the study periods, with an increase in the proportion of women in the 1994–2002 periods. CD4 cell counts at cohort enrollment have increased over the years and in the last period of the study over one third of the patients have CD4 counts at cohort enrollment higher than 350 cells/mm^3^ (Table1). Moreover, when analyzing the median of CD4 counts of all patients under follow-up at each study period, we noticed that it had progressively increased over the years, reaching 458 cells/mm^3^ in the latter period of the study (Figure 1). The use of antiretroviral therapy also increased over the years, and in the most recent period, almost 80% of the patients were under cART (Figure 2).

**Figure 3:**
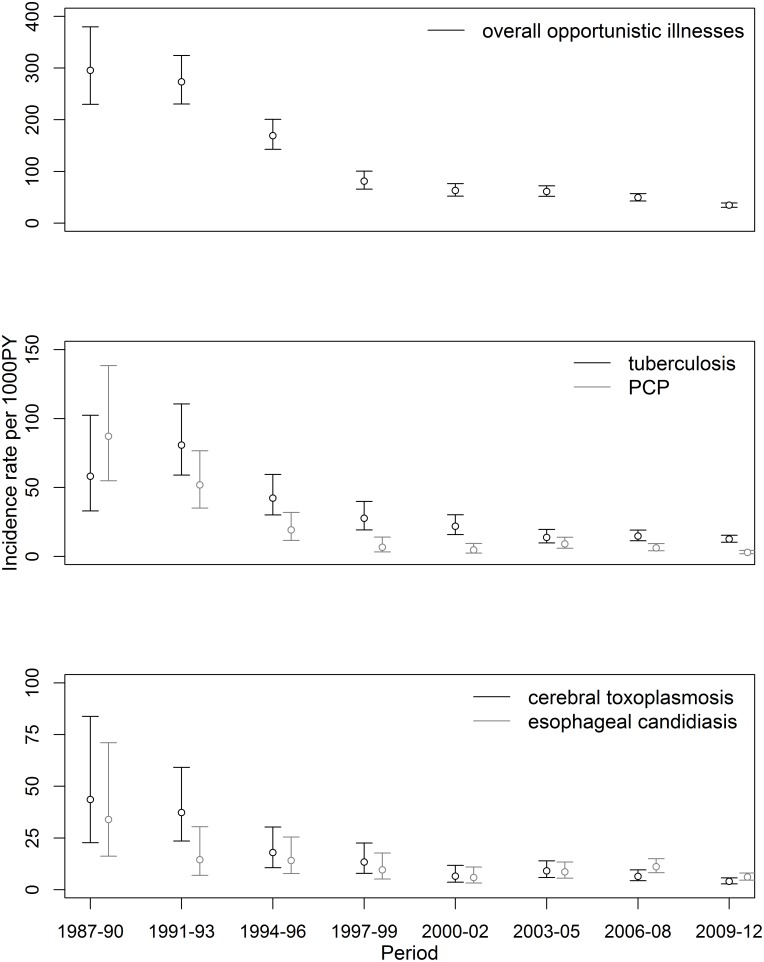
Incidence rates (per 1000 persons-years) and confidence interval for overall opportunistic illnesses, tuberculosis, *Pneumocystis jirovecii* pneumonia, cerebral toxoplasmosis and esophageal candidiasis by calendar period.

During the study period, the incidence rates of overall OIs decreased from 295.4/1000 PY in 1987–1990 to 34.6/1000 PY in 2009–2012, with an incidence rate ratio (IRR) of 0.12 for the period 2009–2012 compared to 1987–1990, p<0.001 (Figure 3, Table 2). Incidence rates for specific OIs have also significantly decreased over the years (Table 2). Tuberculosis, esophageal candidiasis, cerebral toxoplasmosis and PCP had the highest incidence rates during the study period.

**Table 2:** First opportunistic illness after enrollment in IPEC cohort, absolute numbers and incidence rates per 1000 persons-years for 1987–2012 and by calendar period.

	1987–2012		1987–1990		1991–1993		1994–1996		1997–1999		2000–2002		2003–2005		2006–2008		2009–2012		p-valuefor trend^a^	IRR^b^
Person-years	18,137		206		483		780		1,047		1,694		2,317		3,877		7,735			
	n	IR	n	IR	n	IR	n	IR	n	IR	n	IR	n	IR	n	IR	n	IR		
**Overall opportunistic illnesses**	1119	61,70	61	295,44	132	273,47	132	169,27	85	81,21	107	63,18	142	61,30	192	49,53	268	34,65	<0.01	0.12
**Tuberculosis**	336	18.53	12	58.12	39	80.80	33	42.32	29	27.71	37	21.85	32	13.81	57	14.70	97	12.54	<0.01	0.22
**Esophageal candidiasis**	155	8.55	7	33.90	7	14.50	11	14.11	10	9.55	10	5.90	20	8.63	43	11.09	47	6.08	<0.01	0.18
**Toxoplasmosis cerebral**	143	7.88	9	43.59	18	37.29	14	17.95	14	13.38	11	6.50	21	9.07	25	6.45	31	4.01	<0.01	0.09
**Pneumocystis carinii pneumonia**	140	7.72	18	87.18	25	51.79	15	19.24	7	6.69	8	4.72	21	9.07	24	6.19	22	2.84	<0.01	0.03
**Herpes simplex virus**	64	3.53	4	19.37	3	6.22	1	1.28	6	5.73	18	10.63	5	2.16	11	2.84	16	2.07	<0.01	0.11
**Cytomegalovirus**	58	3.20	1	4.84	10	20.72	13	16.67	2	1.91	7	4.13	7	3.02	6	1.55	12	1.55	<0.01	0.32
**Extrapulmonar cryptococcosis**	52	2.87	1	4.84	7	14.50	5	6.41	7	6.69	6	3.54	6	2.59	8	2.06	12	1.55	<0.01	0.32
**Kaposi Sarcoma**	45	2.48	2	9.69	8	16.57	8	10.26	3	2.87	1	0.59	10	4.32	4	1.03	9	1.16	<0.01	0.12
**Crypstosporidiosis**	31	1.71	2	9.69	4	8.29	17	21.80	0	0.00	1	0.59	3	1.30	2	0.52	2	0.26	-	0.03
**Isosporosis**	30	1.65	3	14.53	9	18.65	4	5.13	2	1.91	2	1.18	2	0.86	4	1.03	4	0.52	-	0.04
**Non tuberculosis mycobacterium**	26	1.43	0	0.00	0	0.00	7	8.98	2	1.91	3	1.77	7	3.02	4	1.03	3	0.39	-	0.04^c^
**Disseminated histoplasmosis**	15	0.83	1	4.84	1	2.07	3	3.85	1	0.96	1	0.59	1	0.43	2	0.52	5	0.65	-	0.13
**Non-Hodgkin lymphoma**	14	0.77	1	4.84	0	0.00	1	1.28	2	1.91	1	0.59	2	0.86	1	0.26	6	0.78	-	0.16

Diseases with less than 10 cases reported during the study period are not shown (progressive multifocal leukoencephalopathy n = 8, invasive cervical cancer n = 1, coccidioidomycosis n = 1).

a Trend was tested for all illnesses with 40 cases of more during the study period.

b Incidence rate ratio between 2009–2012 and 19987–1990 periods.

c Reference period was 1994–1996 since the IR in previous periods were equal to zero.

Tuberculosis was the most incident OI, except for the first study period (1987–1990). An incidence rate over 80.8/1000 PY was observed in 1991–1993, and since then the rates have decreased with an IRR of 0.22 (2012–2009 *vs.* 1987–1990, p<0.001) (Figure 3, Table 2). PCP incidence rate was 87.2/1000 PY in 1987–1990, and after that showed the steepest decline over the years, reaching an IRR of 0.03 (2012–2009 *vs.* 1987–1990, p<0.001) (Figure 3, Table 2). Cerebral toxoplasmosis was the third most incident OI in 1987–1990 (incidence rate of 43.6/1000 PY), experienced over the years a significant decrease in its incidence rates (IRR = 0.09, 2012–2009 *vs.* 1987–1990, p<0.001) and currently persists as the third most incident OI, with an incidence rate of 4.0/1000 PY (Figure 3, Table 2). Esophageal candidiasis was the fourth most incident OI in 1987–1990 with an incidence rate of 33.9/1000 PY, its incidence decreased over the years (IRR = 0.18, 2012–2009 *vs.* 1987–1990, p<0.001), and currently it is the second most incident OI (6.1/1000 PY) (Figure 3, Table 2).

## Discussion

In this study, we have shown that the incidence rates of OI decreased over the years, with a trend of reduction that began even before cART was made universally available in Brazil. These results are in agreement with those reported from others cohorts and most likely reflect improvements in the general care of HIV infected patients in addition to the use of specific OI preventive measures as well as mono and dual antiretroviral therapy [21], [26], [31]. The most incident OIs in our casuistic (tuberculosis, esophageal candidiasis, cerebral toxoplasmosis and PCP) are also the most frequent OIs reported in other studies from high-income and low-income settings [2], [4], [6].

Tuberculosis represents the most incident OI in our cohort, with an incidence rate that is currently two-fold higher than the second most incident OI, esophageal candidiasis (12.54/1000 PY vs. 6.08/1000 PY). The 85% reduction observed in tuberculosis incidence rate over the years (1991–1993 *vs.* 2009–2012) is similar to the incidence rate variation observed in multiple cohorts, in both high-income and low-income settings [3], [6], [15], [39], and in our setting it might be a result of overall antiretroviral therapy expansion. In a recently published meta-analysis, the summary estimate of the risk reduction associated with the use of cART across all CD4 counts was 67% (95%CI 61–73) [42]. Randomized clinical trials such as the Cipra HAITI study and the HPTN 052 also showed a 50% reduction after cART initiation among individuals with CD4 cell counts higher than 250 cells/mm^3^ and 350 cells/mm^3^, respectively [16], [41].

HIV and tuberculosis co-infection is a huge challenge for countries with high tuberculosis prevalence, as Brazil, and, in these settings, tuberculosis is still an important cause of severe morbidity [34] and the main cause of death of HIV infected individuals [17], [22], [45]. Unlike others OIs, tuberculosis can occur in a HIV-infected patient independent of the degree of immunodeficiency [3] and the risk of developing tuberculosis is 20 to 37 times higher in HIV infected persons than in those without HIV infection [44].

In our cohort, tuberculosis prophylaxis has been used for HIV infected patients since 1994 according to national guidelines [28]. In the guidelines, isoniazid prophylaxis is recommended for HIV-infected patients with positive tuberculin skin test (TST) and no history of previous tuberculosis [12]. Previous studies have already shown the protective effect of isoniazid prophylaxis on tuberculosis incidence, independently of cART use [1], [14], [15], particularly for those patients with positive TST [32]. Despite the fact that a meta-analysis did not find a reduction in tuberculosis incidence in TST negative patients [1], considering the high risk of false-negative results (up to 66%) in HIV infected patients [7] and operational barriers related to TST implementation and realization, the WHO recommends that in resource-constrained settings TST should not be a requirement for initiating isoniazid prophylaxis [44]. Given that operational barriers and late presentation of HIV-infected patients (that can be associated to false negative TST results) prevail in our cohort, it is possible that isoniazid prophylaxis has been underused, resulting in a high incidence of tuberculosis, even in the most recent periods. Moreover, a recent study conducted in several HIV clinics in Rio de Janeiro showed that efforts to improve coverage of isoniazid prophylaxis to HIV infected patients by training clinicians in tuberculosis prevention resulted in a significant decrease in tuberculosis incidence [14]. It is clear that efforts to properly address the tuberculosis and HIV co-infection burden in our setting are urgently needed.

Cerebral toxoplasmosis was the third most incident OI in our cohort, with and incidence rate that decreased from 43.6/1000 PY in 1987–1990 to 4.0/1000 PY 2009–2012. Both in pre and post cART era, cerebral toxoplasmosis incidence rates observed in our cohort are higher than those observed in other studies both from high and low-middle income countries [23], [35], [37], [38]. Brazilian prevalence of *Toxoplasma gondii* infection is high [13], and specifically in the HIV-infected population this prevalence can reach 80% [46]. A significantly lower Toxoplasma seroprevalence has been estimated in HIV cohorts from high-income settings (11–16%) [20], [24]. It is likely that the elevated incidence rates of cerebral toxoplasmosis observed in our cohort is a result of the high burden of toxoplasmosis infection in Brazil.

PCP was the most incident OI in the first period of our study, with incidence rates comparable to those observed in high-income cohorts in pre cART era [25], [40]. The decreasing trend in the incidence rate of PCP likely results from the use of chemoprophylaxis and antiretroviral therapy. Currently PCP incidence rate in our cohort is lower than that observed in both in high income and low-middle income settings in the post cART era [10], [23], [25], [33], [35].

Esophageal candidiasis incidence rates decreased since the first period of the study, and remained relatively stable after the 2000–2002 period. The rates observed in our study pre and post cART availability are similar to those from high and middle-low income settings [6], [25], [35], and as well as its incidence rates stabilization observed after 2002 [6].

There are limitations and strengths to our study that should be considered. The clinical database for the cohort is based in chart-abstracted information and thus subject to errors in the process of collecting the data from the medical charts. To address this concern and examine the degree of this limitation, a clinical database validation and chart review was performed and great consistence (95%) between the database and original medical charts was found. Another point worth mentioning is the possible underestimation of OI events due to seek of care elsewhere. However, IPEC provides general multidisciplinary care including inpatient care free of charge with an emergency entrance available and, as such, the likelihood that they resort to IPEC in case of illness is high thus minimizing this potential bias.

In conclusion, the incidence rates of OIs overall and of specific illnesses decreased over the years but they still occur in an unacceptable frequency. Tuberculosis and HIV co-infection represent a challenge for the health care professional and policy makers in places with high tuberculosis burden such as Brazil. Continued efforts to address the tuberculosis-HIV co-infection remain highly relevant and needed. We found that cerebral toxoplasmosis incidence in our setting was higher than that of other settings suggesting that the baseline burden of tropical infectious diseases in a particular place influences the incidence of the disease among the HIV infected population. Knowledge of the current burden of infectious diseases among the HIV and non-HIV infected can help plan control efforts for both populations.
